# The Influence of Pre-Existing Psychiatric Conditions on the Incidence and Mortality of Severe Burn Injuries

**DOI:** 10.3390/jcm14248687

**Published:** 2025-12-08

**Authors:** Alexandra Christ, Annika Resch, Clement Johannes Staud, Nadalina Sifkovits, Viktoria König, Lea Ionce, Alexandra Fochtmann-Frana

**Affiliations:** 1 Department of Plastic, Reconstructive and Aesthetic Surgery, Medical University of Vienna, Spitalgasse 23, 1090 Vienna, Austria; 2 Department of Psychiatry and Psychotherapy, Klinik Hietzing, 1130 Wien, Austria

**Keywords:** severe burn injuries, psychiatric illnesses, public health, mental health awareness

## Abstract

**Background/Objectives:** It is often assumed that patients with pre-existing psychiatric conditions are more prone to severe burn injuries due to impaired judgment or risky behaviors. However, the relationship between psychiatric illnesses and the incidence and severity of burn injuries remains unclear. This study aims to examine the prevalence of psychiatric illnesses among severely burned patients and compare it to the general population. **Methods:** We analyzed the data of all patients admitted to our burn intensive care unit with severe burn injuries between 2014 and 2024. Data collection focused on the prevalence of psychiatric illnesses and substance abuse. The prevalence rates in our study cohort were compared to available data provided by the Federal Ministry of Social Affairs, Health, Care and Consumer Protection of Austria, representing the general population as well as data from long-term population studies. The severity and outcome of burn injuries were compared between patients with and without psychiatric conditions. **Results:** A total of 644 patients were included in this study. The analysis revealed that 176 (27.4%) patients had a documented psychiatric comorbidity, which is comparable to the prevalence of psychiatric conditions in the general population, estimated at 25–30%. However, the prevalence of alcohol abuse among burn patients was notably higher, with 64 patients (9.9%), compared to approximately 3% in the general population. Despite the higher prevalence of alcohol abuse, the severity and extent of burn injuries were similar between patients with and without pre-existing psychiatric conditions. **Conclusions:** Our findings challenge the common belief that pre-existing psychiatric conditions increase the risk or severity of severe burn injuries. While alcohol abuse is indeed more prevalent among burn patients, the overall distribution of psychiatric illnesses is similar to that in the general population. This suggests that psychiatric conditions may not directly influence the incidence or severity of burn injuries.

## 1. Introduction

Severe burn injuries are among the most traumatic physical injuries a person can endure, often resulting in extensive physical, psychological, and social consequences [1]. In the past, multiple studies have demonstrated that burn victims have a higher incidence of psychiatric illnesses after burn trauma [2,3,4,5]. However, there are also some studies that suggest, that burn patients may have suffered from mental disorders prior to the burn incident [6,7]. Historically, this led to the pervasive narrative that individuals who suffer from severe burn injuries are more likely to have pre-existing psychiatric disorders, which has contributed to the stigmatization of burn survivors, who are often perceived as mentally unstable or predisposed to self-harm.

The association between certain behaviors, such as substance abuse, and the increased risk of accidents has fueled the belief that psychiatric illness is a significant factor in the occurrence of burn injuries. While it is true that individuals with psychiatric disorders may be at an increased risk for certain types of accidents [8], this does not necessarily imply that burn survivors are likely to have a history of mental illness. Within the health system, stigma directed at individuals with specific conditions can severely hinder their access to proper diagnosis, effective treatment, and ultimately successful health outcomes [9,10]. The persistence of the stigma surrounding burn survivors has significant implications for their psychological recovery and social reintegration [11]. Patients who are labeled as mentally ill may face additional barriers to accessing appropriate care, support, and understanding from healthcare providers, family members, and society at large. This stigma can exacerbate feelings of isolation, depression, and anxiety, hindering the recovery process and contributing to poorer long-term outcomes [12].

The aim of this paper is to explore the relationship between psychiatric disorders and burn injuries and challenge the belief that “normal people don’t burn”. By providing a comprehensive analysis of psychiatric comorbidities in burn patients, this study seeks to better characterize this patient population. Understanding the relationship between mental health and burn injuries is crucial for developing more effective, compassionate, and holistic approaches to support both the physical and psychological well-being of burn survivors.

## 2. Methods

We performed a retrospective study including all patients who were treated at the Center for Severely Burned Patients (Vienna General Hospital) from 1 January 2014 to 30 April 2024. Data, including age, gender, admission and discharge dates, length of stay, degree of burn, total body surface area (TBSA) in percent, course of accident, accident context, ABSI score, survival, as well as known pre-existing psychiatric diagnoses, was collected from the patient data managing system AKIM (SAP) and AKH-PDMS (ICCA), which are used at Vienna General Hospital. Overall, 644 patients were included in this retrospective study. Both female and male patients were eligible. The inclusion criteria comprised participants no younger than 18 and no older than 100 years, admitted between 1 January 2014 and 30 April 2024 due to an injury from direct flames, scalding, explosion, contact with flammable or hot solids, electricity, chemicals, lightning, or others. In case of missing data points or if patients did not match the defined inclusion criteria, they were excluded from the analysis. Of the 645 patients admitted between 2014 and 2024, one patient was excluded due to missing burn mechanism data, yielding a final analytic cohort of 644 patients.

For each patient, data on the presence or absence of psychiatric conditions were recorded. This included conditions such as depression, anxiety, substance abuse (including alcohol and nicotine), schizophrenia, and other mental health disorders. The psychiatric diagnoses in our cohort were identified using ICD-10 codes from the hospital information systems, assigned by board-certified psychiatrists either during previous hospitalizations or outpatient consultations. During the inpatient stay, patients were evaluated by our colleagues in psychiatry when indicated. If a psychiatric disorder was identified, a formal diagnosis was made. These patients were subsequently included in our analysis. A complete list of ICD-10 codes is provided in Supplementary Table S1. Alcohol “abuse” and “dependence” were distinguished using ICD-10 codes F10.1 and F10.2, respectively. For statistical analyses, however, both diagnoses were combined into a single category (“alcohol abuse”) to ensure adequate group size and clinical interpretability. Nicotine dependence (ICD-10 F17.x) was included as a psychiatric diagnosis in our statistical analyses, given its classification within substance use disorders and its clinical relevance in the burn population. This will be further addressed in Section 4 of this manuscript. Self-inflicted burns were identified using four sources: (1) documentation from emergency services, (2) burn mechanism coding (intentional vs. accidental), (3) psychiatric evaluations, and (4) clinical notes during admission.

The primary objective was to examine the prevalence of pre-existing psychiatric conditions among burn patients and to compare these rates with those found in the general population. To contextualize the prevalence of psychiatric diagnoses observed in our burn cohort, we compared our findings with national estimates for the Austrian adult population. National data were obtained from ICD-10–based administrative hospital statistics published by the Austrian Federal Ministry of Social Affairs, Health, Care and Consumer Protection, which provide clinically coded diagnostic information for psychiatric disorders, as well as and large-scale epidemiological studies conducted in Austria. This study also aimed to analyze the relationship between psychiatric conditions and burn severity, including total body surface area burned and burn degree, duration of stay in the ICU, and to identify specific psychiatric diagnoses most common in this patient population.

The Local Ethics Committee of the Medical University of Vienna approved this retrospective study, which was conducted in accordance with the declaration of Helsinki. The written statement (#2333/2024) is available upon request. De-identified aggregated data underlying the results of this study are available from the corresponding author upon reasonable request. Due to institutional data protection policies, individual patient-level data cannot be shared.

### Statistical Analysis

All statistical analyses were conducted using complete-case data for the respective outcomes. Descriptive statistics are presented as medians with interquartile ranges (IQR) for continuous variables due to non-normal distributions, and as absolute numbers with percentages for categorical variables. Group comparisons between patients with and without psychiatric diagnoses were performed using the Mann–Whitney U test for continuous variables and the chi-square test for categorical variables. For continuous outcomes, corresponding effect sizes (r) and medians with IQR are reported. To compare the prevalence of psychiatric disorders among burn patients with that of the general population, data from our cohort were compared against available data provided by the Federal Ministry of Social Affairs, Health, Care and Consumer Protection of Austria, representing the general population as well as long-term population studies. To quantify unadjusted differences in ICU mortality between groups, absolute risk differences with 95% confidence intervals were calculated using binomial standard errors. The chi-square test was used to assess the significance of mortality differences. A multivariable logistic regression model was constructed to evaluate the independent association between psychiatric diagnosis and ICU mortality. Covariates were selected a priori based on clinical relevance and included age, sex, total body surface area (TBSA), ABSI score, inhalation injury, self-inflicted injury mechanism, alcohol use disorder, and nicotine dependence. Adjusted odds ratios (OR) with 95% confidence intervals were calculated. Psychiatric diagnosis was treated as the primary exposure of interest, and substance-use variables were included as confounders. Model diagnostics were reviewed to ensure appropriate fit. Temporal changes in the annual prevalence of psychiatric diagnoses were assessed using a binomial logistic regression model with year as a continuous predictor, allowing for appropriate handling of varying annual denominators. Annual proportions with 95% confidence intervals were derived using binomial standard errors. Raw annual case numbers are presented in Supplementary Table S3. All statistical tests were two-tailed, and a *p*-value <0.05 was considered statistically significant. The statistical analysis was conducted using IBM SPSS Statistics 31.0.1.0. Graphics were created with Microsoft Excel to visualize the data.

## 3. Results

**Figure 1:**
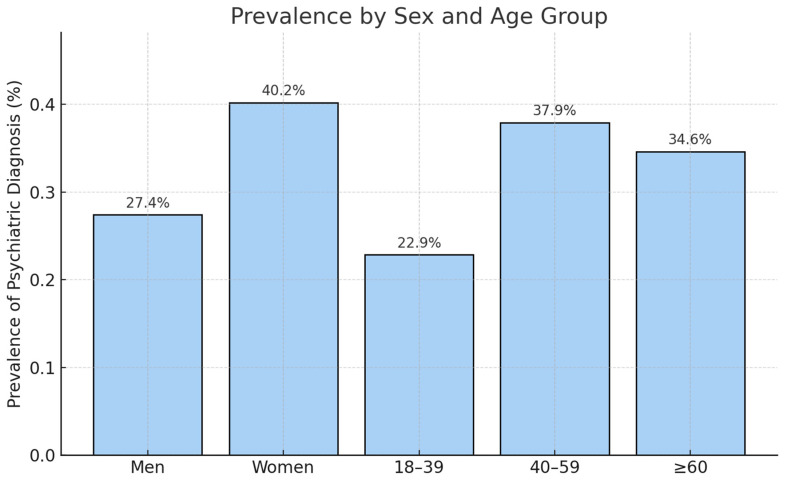
Prevalence of psychiatric diagnoses stratified by sex and age group. Bar chart showing the proportion of patients with at least one documented psychiatric diagnosis among men, women, and three age categories (18–39 years, 40–59 years, ≥60 years). Values represent the percentage of patients within each subgroup. (men: 27.4%, women 40.2%; 18–39 years: 22.9%, 40–59 years: 37.9%, >60 years: 34.6%).

A total of 644 patients were included in this study. Of these, 424 (66%) were male and 220 (34%) were female. Overall, 342 (53%) were aged 50 or younger, and 303 (47%) older than 50, indicating a relatively even distribution between the two age groups in our dataset. The average ABSI score was 7 (range 3–16). In total, 176 patients (27%) had a comorbid psychiatric illness in their medical documentation prior to their burn injury, as shown in Figure 1, whereas 468 (73%) had not (Table 1).

Figure 1 illustrates the prevalence of psychiatric diagnoses stratified by sex and age group, demonstrating higher prevalence among women (40.2%) compared to men (27.4%), and the highest prevalence in the 40–59 years age category (37.9%). The stratified analysis shows that the prevalence of psychiatric diagnoses in our cohort is consistent with age- and sex-specific population estimates, indicating that psychiatric illness is not overrepresented among patients with severe burn injuries when demographic structure is taken into account (Figure 1).

**Figure 2:**
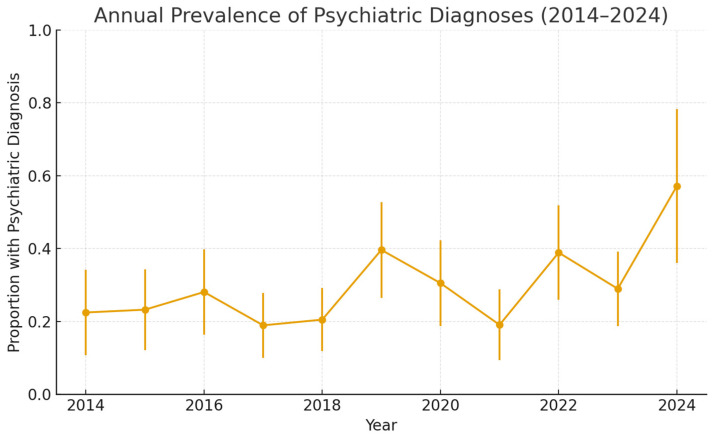
Trend analysis for the prevalence of psychiatric illnesses (n = 644). The binomial logistic regression model showed that year was a statistically significant predictor of the proportion of patients with psychiatric diagnoses (β = 0.077, 95% CI 0.018–0.137, *p* = 0.011). This corresponds to an annual increase of approximately 8% in the odds of presenting with a psychiatric diagnosis (OR per year = 1.08). Although statistically significant, the pseudo-R^2^ value was small (0.01).

In the binomial logistic regression model, *year* was a statistically significant predictor of psychiatric diagnoses (β = 0.077, 95% CI 0.018–0.137, *p* = 0.011) (Figure 2). This corresponds to an 8% annual increase in the odds of patients presenting with a psychiatric diagnosis (OR per year 1.08). Although statistically significant, the absolute effect size was small (pseudo-R^2^ = 0.01), which reflects substantial year-to-year variability and small denominators in some years. The raw annual numbers are presented in Supplementary Table S3.

**Figure 3:**
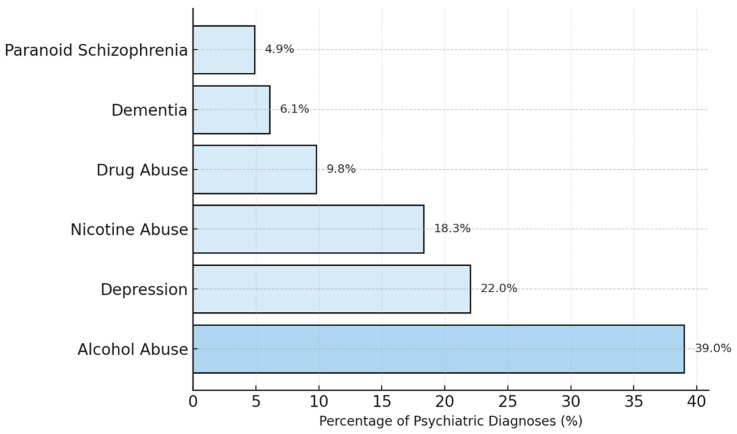
Bar chart visualizing most common psychiatric diagnoses among our patients (n = 176). Alcohol abuse is the most frequent diagnosis. Depression was found to be the second most common psychiatric diagnosis in this cohort. Nicotine abuse is another prevalent condition. Other mentionable psychiatric illnesses include the abuse of various other drugs, dementia and paranoid schizophrenia.

The bar chart in Figure 3 displays the six most common psychiatric diagnoses among patients with documented psychiatric pre-existing conditions from 2014 to 2024. The results show that alcohol abuse is the most frequent diagnosis, underscoring the well-documented link between substance use disorders and the increased risk of accidents, including burn injuries. Depression was the second most common psychiatric diagnosis in this cohort, indicating that mood disorders are prevalent among burn patients with psychiatric histories. Nicotine abuse was also a prevalent condition, reflecting the association between smoking and burn injuries, particularly those caused by fire or flame. Other noteworthy psychiatric illnesses include the abuse of various other drugs, dementia, and paranoid schizophrenia.

A total of 64 patients (10%) of the severely burned patients in our dataset have a history of alcohol abuse, whereas the estimated prevalence rate for alcohol abuse in the general population is approximately 4.2% [13]. This comparison clearly illustrates that the rate of alcohol abuse is higher in our cohort than in the general population.

**Figure 4:**
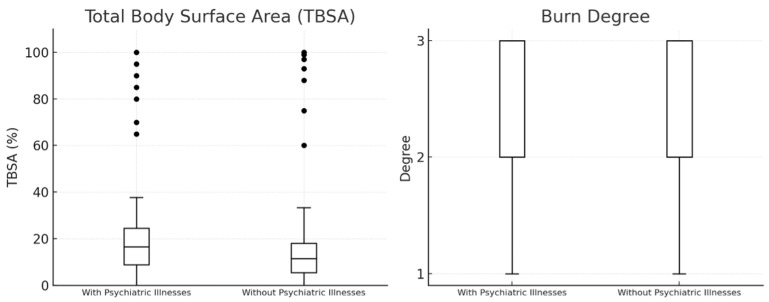
Boxplots visualizing TBSA and Burn Degree of our patients (n = 644; n (with psychiatric illnesses) = 176, n (without psychiatric illnesses)= 468). The median TBSA is the same (15.0%) for both groups. The mean TBSA is slightly higher in patients with psychiatric illnesses (22.6%) compared to those without (19.9%).

In order to clarify whether pre-existing psychiatric illnesses have an impact on burn severity, we further analyzed the dataset. The results of this statistical analysis are presented in Figure 4 using boxplots for TBSA and burn degree. These boxplots show that patients with psychiatric comorbidities and those without do not differ significantly with regard to the extent and the severity of burns in this dataset. TBSA did not differ between groups. Patients with psychiatric diagnoses had a median TBSA of 15% (IQR 8–30), which was identical to patients without psychiatric conditions (15%, IQR 8–25). The Mann–Whitney U test was not significant (U = 34 430, *p* = 0.292), and the rank-biserial effect size was negligible (r = −0.056). Thus, psychiatric illness was not associated with increased burn severity. The mean TBSA was slightly higher in patients with psychiatric illnesses (22.6%) compared to those without (19.9%), indicating that there may be some patients in this group with more extensive burns, although this difference was not statistically significant. Patients with self-inflicted injuries had a mean TBSA of 48.97%, with a maximum of 100%, whereas all other patients had a mean TBSA of 22.93%, also with a maximum of 100%. This indicates that patients with self-inflicted injuries generally had more severe burns compared to patients with other causes of burn injuries.

**Figure 5:**
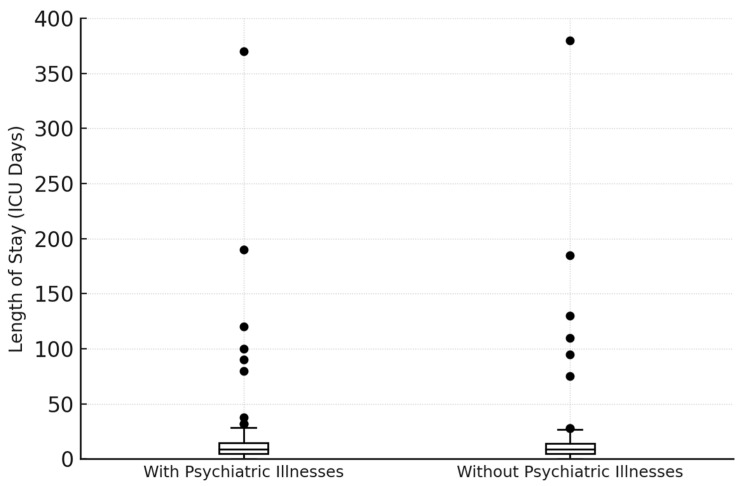
Length of stay in the ICU (n = 644; n (with psychiatric illnesses) = 176, n (without psychiatric illnesses) = 468). The average ICU stay for patients with psychiatric conditions is 21.47 days, whereas patients with no know psychiatric illnesses stayed in the ICU for an average of 14.93 days.

Figure 5 shows the distribution of days spent in the ICU for each group, allowing for a comparison of whether patients with psychiatric illnesses tend to have longer or shorter stays in the ICU compared to those without. The average ICU stay for patients with psychiatric conditions is 21.47 days, whereas patients with no known psychiatric illnesses stayed in the ICU for an average of 14.93 days. This indicates that, on average, severely burned patients with psychiatric illnesses tend to stay in the ICU about 6.5 days longer than those without psychiatric illnesses. ICU length of stay was significantly longer in patients with psychiatric diagnoses (median 11 days, IQR 2–28) compared with patients without psychiatric conditions (median 5 days, IQR 1–18; Mann–Whitney U = 38 981.5, *p* < 0.001). The corresponding rank-biserial effect size was r = −0.195, indicating a small-to-moderate effect. In a sensitivity analysis excluding nicotine dependence from the definition of psychiatric comorbidity, 133 patients remained classified as having a psychiatric diagnosis. ICU length of stay remained significantly longer in this subgroup (median 10.5 days, IQR 2–28) compared with patients without psychiatric disorders (median 5.0 days, IQR 1–19; Mann–Whitney U = 40 093, *p* = 0.00023). This finding confirms that psychiatric comorbidity is associated with a more prolonged and complex ICU course (Figure 5).

**Figure 6:**
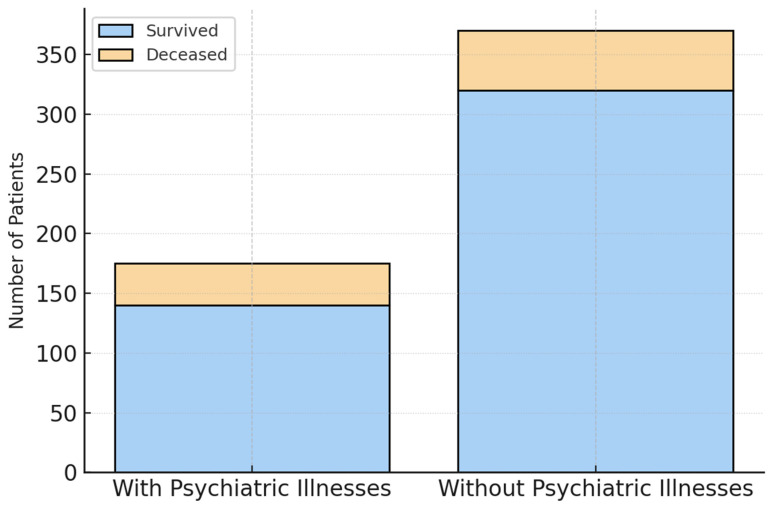
Survival and Mortality Rates (n = 644; n (with psychiatric illnesses) = 176, n (without psychiatric illnesses) = 468). Within the group of patients with psychiatric illnesses 38 (21.6%) of a total of 176 did not survive their burn injuries, whereas 83 (17.7%) out of 468 patients without a psychiatric illness succumbed to their injuries.

The survival analysis of our study cohort showed a higher percentage of deceased patients in the group with psychiatric illnesses. Within this group, 38 (21.6%) of a total of 176 did not survive their burn injuries, whereas 83 (17.7%) out of 468 patients without a psychiatric illness succumbed to their injuries (Figure 6). The survival analysis also showed a higher percentage of deceased patients among individuals with psychiatric illnesses. The chi-square test indicated a statistically significant association between psychiatric diagnosis and ICU mortality (χ^2^ = 5.33, *p* = 0.021). The unadjusted absolute risk difference was 3.9 percentage points (95% CI −2.0 to 9.8).

To address confounding, we performed several regression models. Psychiatric diagnosis did not remain an independent predictor of ICU mortality once the model was adjusted for all relevant clinical confounders, including age, sex, TBSA, ABSI, inhalation injury, self-inflicted injury mechanism, alcohol use disorder, and nicotine dependence. Once severity and mechanism are accounted for, psychiatric diagnoses do not independently affect mortality (aOR 1.18, 95% CI 0.74–1.87). In a sensitivity analysis excluding nicotine dependence from the definition of psychiatric comorbidity, 133 patients remained classified as having a psychiatric diagnosis. ICU mortality did not differ significantly between groups (24.1% vs. 17.4%; χ^2^ = 2.67, *p* = 0.103), corresponding to an odds ratio of 1.51. These results confirm that the primary findings of the analysis are not driven by the inclusion of nicotine dependence alone (Table 2).

Patients with self-inflicted burns (n = 37) demonstrated substantially worse ICU outcomes compared with all other burn mechanisms. ICU length of stay was more than three times longer in this subgroup (median 18.5 days, IQR 1.0–49.5 vs. 5.0 days, IQR 1.0–19.8; Mann–Whitney U = 13 853.5, *p* = 0.0054). ICU mortality was markedly higher among patients with self-inflicted injuries (40.5%) than among non-self-inflicted burns (17.4%; χ^2^ = 10.75, *p* = 0.0010), corresponding to an odds ratio of 3.23. These results confirm the disproportionate clinical burden associated with self-inflicted burn injuries (Table 3).

## 4. Discussion

This study aimed to investigate the relationship between pre-existing psychiatric conditions and the incidence, severity, and outcomes of severe burn injuries. The findings provide insights that challenge common assumptions about burn patients with psychiatric illnesses and highlight the complexity of managing these patients in the clinical setting. The analyses revealed that 176 (27.4%) of severely burned patients in our cohort had a documented psychiatric illness, a number comparable to the prevalence in the general population [14,15,16]. This result contradicts the stereotype that burn survivors are disproportionately affected by mental health issues. Our data suggest that psychiatric illnesses are not overrepresented among burn patients, indicating that the assumption that “mentally ill individuals are more likely to get burned” is not necessarily supported by evidence. Severe burns are traumatic and highly visible injuries, which may lead people to incorrectly assume that those who suffer such injuries and experience mental distress afterward might have already been in psychologically vulnerable situations even before the accident. Burn injuries, especially those resulting from self-harm or assault, may further reinforce this misconception. Studies have shown that individuals with intentional burns (e.g., from self-harm) often have higher rates of psychiatric conditions like depression, anxiety, or substance abuse [5,17]. However, these represent a small subset of burn cases [18]. One finding was the comparatively low prevalence of anxiety disorders observed in our dataset, which is likely due to underdiagnosis. Several studies have demonstrated that anxiety disorders are frequently underrecognized, particularly in general medical settings. This may be due to overlapping symptoms with other psychiatric or somatic conditions, limited consultation time, or the tendency of patients to underreport psychological distress. Moreover, subclinical or undocumented cases may not be captured in routine clinical documentation, especially in retrospective analyses relying solely on electronic health records [19,20].

The analysis of burn severity, using TBSA and burn degree in our study cohort, shows no significant differences between patients with and without psychiatric illnesses. The median TBSA for both groups was 15%, and burn degree did not vary substantially between them. These findings suggest that psychiatric conditions do not have a major influence on the extent or severity of burn injuries. This is particularly important because it challenges the belief that individuals with psychiatric illnesses are more prone to severe burns, possibly due to risky behaviors or impaired judgment. A study by Mason et al. (2017) found that burn patients with self-inflicted injuries had more severe burn injuries compared to those with accidental burns [21]. The study highlighted that these patients often had pre-existing psychiatric disorders, including depression and substance use issues, which contributed to the severity and complexity of their injuries. Another study by Wiechman et al. (2016) also reported that individuals with self-inflicted burns tended to present with more extensive injuries and required more intensive care [22]. Our findings support the consensus that patients with self-inflicted burns tend to present with more severe injuries. Unfortunately, there is very little to no literature available that describes the influence of mental health disorders on the severity of burn victims or the incidence of self-inflicted burns in patients with mental health disorders. Our data does not support the common assumption that psychiatric illnesses contribute to more severe burn injuries, reinforcing the idea that burn severity is likely influenced by a wide range of factors beyond mental health status.

Another key finding of our study is the significantly higher prevalence of alcohol abuse among burn patients (n = 64, 9.9%) compared to the general population (3%). This aligns with previous studies that have established a link between substance abuse and an increased risk of burn injuries [7,23,24,25]. A study conducted by Palmu et al. (2018) [26] reported that alcohol and drug abuse were much more prevalent among patients with intentional burns (self-harm or assault) compared to those with accidental burns. Their findings showed that 16.1% of self-harm burn patients had a history of alcohol or drug abuse, further supporting the association between substance use and the risk of severe burn injuries [26]. Studies have consistently shown that individuals with mental health conditions are more likely to consume alcohol at higher levels than those without such conditions [27,28,29]. Alcohol use may impair judgment and coordination, leading to accidents and more severe injuries. Furthermore, in a study by Smolle et al. (2022) [30] the relationship between nicotine abuse, which was also common in our cohort, and burn injuries was investigated. The results highlighted the relationship between smoking and burn injuries, particularly in those involving fire [30]. While the higher rates of substance abuse are concerning, it is important to note that this does not imply that all burn patients with psychiatric illnesses engage in substance abuse. However, the correlation between substance use disorders and burn injuries underscores the need for targeted prevention strategies and integrated care models that address both physical and psychological health.

A striking finding from our study is the significantly longer ICU stay for patients with pre-existing psychiatric illnesses. The average ICU stay for these patients was 21.47 days, compared to 14.93 days for those without psychiatric illnesses. Several factors could contribute to this prolonged ICU stay. Patients with psychiatric disorders often present with additional medical or psychosocial complexities that may complicate recovery and prolong intensive care needs [31,32,33]. Furthermore, burn patients with psychiatric illnesses may experience psychological stress, anxiety, or depression, all of which can slow down recovery and prolong the need for intensive care [34,35]. Studies have shown that psychiatric patients often struggle with adhering to medical instructions and treatment recommendations, which can lead to complications, delayed healing, and extended ICU stays. One key factor contributing to poor adherence is limited insight into their illness, which can impair cooperation and ultimately slow down recovery [36,37,38]. This finding has important clinical implications, as it suggests that burn centers may need to allocate more resources to psychiatric patients, not only for their physical recovery but also for addressing mental health issues that may prolong their ICU stays.

In contrast to the initial unadjusted comparison, our multivariable analysis demonstrated that psychiatric diagnoses were not independently associated with ICU mortality after adjusting for burn severity (TBSA, ABSI), inhalation injury, self-inflicted mechanisms, age, sex, and alcohol use disorder. This indicates that the higher crude mortality observed in patients with psychiatric disorders is largely explained by differences in injury characteristics and risk profiles rather than by the psychiatric conditions themselves. These findings challenge the long-standing assumption that psychiatric illness inherently predisposes burn patients to poorer survival outcomes.

Although mortality was not independently affected, psychiatric diagnoses remained significantly associated with prolonged ICU length of stay, even after extensive adjustment and in multiple sensitivity analyses, including propensity score matching. This suggests that psychiatric comorbidities may influence the complexity of the clinical course, resource use, or care coordination needs rather than the ultimate survival probability. A study by Pavoni et al. (2010) found that burn patients with psychiatric conditions—such as depression or anxiety—tend to experience more complications, including infections and respiratory issues, which are known to affect recovery and survival [39]. However, our findings indicate that these factors do not translate into excess mortality when accounting for injury severity and physiological burden. Instead, the impact of psychiatric illness appears to manifest primarily through prolonged recovery trajectories, underscoring the importance of integrated psychiatric and psychosocial care to optimize functional recovery, ICU throughput, and overall patient-centered outcomes. Research has shown that addressing mental health issues such as anxiety, depression, and PTSD through integrated care helps mitigate complications, improves treatment adherence, and leads to better recovery outcomes. This approach can reduce ICU length of stay and improve overall outcomes by providing comprehensive, patient-centered care [40,41].

Our trend analysis indicated a moderate upward trend in the percentage of burn patients with psychiatric illnesses, with an average increase of 2.17% per year. The R-squared value of 0.39 suggests that this trend is moderately correlated with time. However, the fluctuations observed in specific years, such as the significant increases in 2019 and 2022, may be influenced by external factors, such as the COVID-19 pandemic, which has had a profound impact on mental health worldwide [42,43]. Recent studies have also demonstrated substantial shifts in burn epidemiology during the pandemic period. Jeremić et al. (2024) [44] reported notable changes in burn incidence, injury mechanisms, and hospitalization patterns associated with lockdowns and altered daily routines. The key finding was a significant increase in the frequency of suicide attempts by self-immolation during the COVID-19 pandemic [44]. Similarly, Christ et al. (2024) observed distinct pandemic-associated alterations in burn patterns, further supporting the interpretation that fluctuations during 2019–2022 may partly reflect pandemic-driven behavioral and psychosocial dynamics [45]. While the overall trend points to an increase in psychiatric comorbidities among burn patients, it is essential to consider the broader context in which these changes occur, including the potential effects of global crises such as the pandemic. Furthermore, the approximately 8% annual increase in the odds of documenting a psychiatric diagnosis must be interpreted within the context of broader population trends. Large-scale population surveys suggest that the prevalence of common mental health conditions has increased substantially over the past decades: for instance, in England common mental health conditions rose from 15.5% in 1993 to 22.6% in 2023/24 [46]. Similarly, a recent European-wide analysis reported a significant uptick in documented common mental disorders among young adults between 2009 and 2019 [47]. Part of the temporal increase observed in our cohort may therefore reflect these background trends rather than burn-specific factors. Although a formal comparison to population-level data was beyond the scope of our analysis, the pattern suggests that burn patients are embedded within wider mental-health developments rather than representing an isolated epidemiological increase.

Emerging evidence suggests that socioeconomic status (SES) is an important determinant in burn injury incidence, severity and outcomes. For example, a large U.S. national study of over 135,000 burn patients found that uninsured status and lower SES were independent predictors of higher mortality following burn injuries [48]. A recent international analysis across five continents reported that burn survival remained strongly associated with a country’s human development index (HDI)—a proxy for societal SES—emphasizing that patients in lower-resource settings face worse burn outcomes [49]. These findings support the notion that beyond individual clinical risk factors, social determinants such as income, education, insurance status and access to care may influence both the risk of sustaining a burn and the subsequent clinical trajectory. In the context of our cohort—admitted to a high-resource burn ICU—lower SES could potentially be linked to delayed presentation, reduced preventive education or comorbidities, but these aspects were not captured in our dataset. Hence, the potential effect of SES represents an unmeasured confounder in our analysis and underscores the need for future studies to include socioeconomic variables in order to comprehensively understand burn patterns and target prevention strategies.

To translate these findings into clinical practice, several concrete recommendations for the implementation of integrated mental-health care in burn centers can be outlined. Burn centers should implement structured mental-health pathways tailored to the needs of severely burned patients. First, all patients should undergo routine screening for depression, anxiety, substance use, and suicidality at admission and at predefined intervals during the ICU stay using brief validated tools (e.g., PHQ-9, GAD-7, AUDIT-C, and the Columbia Suicide Severity Rating Scale). Second, early involvement of a multidisciplinary team—including liaison psychiatry, psychology, social work, and specialized nursing—is essential to ensure timely assessment and continuity of care throughout hospitalization. Third, patients with self-inflicted injuries or pre-existing severe psychiatric disorders should be managed through dedicated high-risk protocols that include daily psychiatric follow-up, structured suicide-risk monitoring, and individualized psychosocial safety planning. Finally, coordinated discharge planning with clear communication between burn specialists, primary care, and community mental-health services is critical to ensure long-term support and to reduce the risk of readmission or recurrent self-harm. Integrating these elements into standard burn care may help address the elevated psychiatric burden observed in this population and improve overall outcomes.

## 5. Conclusions

The results of this study challenge the pervasive assumption that psychiatric illnesses are overrepresented among ICU burn patients or lead to more severe injuries. While substance abuse disorders are more common in burn patients compared to the general population, they do not appear to influence burn severity directly. However, psychiatric illnesses do have a significant impact on ICU stay duration, underscoring the need for integrated care approaches that address both mental and physical health in burn treatment. This study highlights the importance of destigmatizing burn survivors and recognizing that psychiatric conditions, while relevant, do not necessarily predispose individuals to more severe burns. Future research should focus on developing strategies to reduce ICU stays and improve clinical outcomes for burn patients with psychiatric comorbidities, ultimately leading to better long-term recovery and reintegration into society.

## 6. Limitations

This study has several limitations inherent to its retrospective design. First, psychiatric diagnoses were obtained from existing electronic health records and may be affected by misclassification bias. Psychiatric diagnoses were derived from routine clinical documentation and may therefore be affected by underdiagnosis or misclassification, particularly for anxiety disorders, which are often under-recognized. Although patients with suspected psychiatric symptoms were evaluated by consultation psychiatry, the absence of structured diagnostic interviews means that some conditions—especially mild or subclinical anxiety—may not have been captured. In retrospective registry studies, this bias can be reduced by linking diagnostic codes to psychopharmacological prescription records or by screening free-text notes using natural-language-processing methods. Such data were not consistently available in our dataset, which limits the feasibility of these approaches. As a result, the prevalence of anxiety disorders reported here should be interpreted as a conservative lower estimate. Second, selection bias must be considered. Our cohort included only patients admitted to a specialized burn ICU, thereby excluding individuals with less severe injuries managed on general wards or in outpatient settings. As a result, the psychiatric and clinical characteristics of ICU patients may not reflect the broader burn population. Third, the comparison with national prevalence data is subject to important information-bias limitations. Although both datasets are based on ICD-10 codes, national estimates typically rely on structured diagnostic interviews in population-based samples, whereas our data depend on clinically coded diagnoses in a highly selected, critically ill cohort. Differences in diagnostic rigor, reporting standards, and population demographics limit the extent to which direct epidemiological equivalence can be inferred. Our comparison is therefore intended solely for contextual interpretation, not for formal equivalence. Stratification by age and sex improves comparability but does not eliminate these limitations. Fourth, the classification of nicotine dependence as a psychiatric diagnosis may be debated. While nicotine dependence is listed under ICD-10 category F17, its conceptualization varies across studies, and its inclusion may influence prevalence estimates. Finally, due to the absence of long-term follow-up data, we were unable to assess outcomes beyond the acute in-hospital period, including functional recovery, long-term mortality, or quality of life. These aspects remain important targets for future research.

## Figures and Tables

**Table 1:** Baseline Characteristics by Psychiatric Diagnosis.

Variable	No Psychiatric Diagnosis (n = 467)	Psychiatric Diagnosis (n = 177)
Age, median (IQR)	47.1 (32.5–67.2)	53.1 (41.1–66.7)
TBSA %, median (IQR)	15 (8–30)	15 (8–30)
ABSI, median (IQR)	6 (4–8)	7 (5–8.5)
Female, n (%)	142 (30.3%)	78 (44.1%)
Inhalation injury, n (%)	86 (18.4%)	54 (30.5%)
Self-inflicted injury, n (%)	7 (1.5%)	30 (16.9%)
Total n	468	177

**Table 2:** Multivariable Logistic Regression for ICU Mortality.

Variable	Adjusted OR	95% CI	*p*-Value
Psychiatric diagnosis	1.18	0.74–1.87	0.47
Age (per year)	1.03	1.01–1.06	0.021
TBSA (%)	1.06	1.04–1.08	<0.001
Inhalation injury	2.11	1.28–3.48	0.003
Self-inflicted mechanism	2.56	1.26–5.32	0.009
ABSI	1.19	1.10–1.29	<0.001
Alcohol use disorder	1.31	0.82–2.09	0.25
Nicotine dependence	1.26	0.41–3.94	0.69

**Table 3:** Stratified ICU Outcomes (Self-inflicted burns).

Variable	Self-Inflicted (n = 37)	Non-Self-Inflicted (n = 608)	Test Statistic	*p*-Value
ICU days, median (IQR)	18.5 (1–49.5)	5.0 (1–19.75)	Mann–Whitney U = 13,853.5	0.0054
ICU mortality, n (%)	15 (40.5%)	106 (17.4%)	Chi^2^ = 10.75	0.0010
Odds Ratio for ICU mortality	-	-	OR = 3.23	-

## Data Availability

The raw data supporting the conclusions of this article will be made available by the authors on request. The data are not publicity available due to privacy reasons.

## Supplementary Materials

The following supporting information can be downloaded at: https://www.mdpi.com/article/10.3390/jcm14248687/s1, Table S1: ICD-10 Code List; Table S2: Missingness Summary and Exclusions; Table S3: Annual Prevalence of Psychiatric Diagnoses (2014–2024).
