# Supplementary material for: The Influence of Pre-Existing Psychiatric Conditions on the Incidence and Mortality of Severe Burn Injuries
Source: J Clin Med. 2025 Dec 8;14(24):8687. doi: 10.3390/jcm14248687 (PMC12734089; doi:10.3390/jcm14248687)
Supplement: Supplementary file 1 [file jcm-14-08687-s001.zip › jcm-3990032-supplementary.pdf]

Supplementary Tables:

Supplementary Table S1 – ICD-10 Code List:

Category	ICD-10 Codes
Depression	F32.x, F33.x
Anxiety disorders	F40.x, F41.x
Psychotic disorders	F20–F29
Bipolar disorders	F30–F31
Personality disorders	F60–F69
Alcohol use disorder	F10.1
Alcohol dependence	F10.2
Other substance use disorders	F11.x–F15.x
Nicotine dependence	F17.x
Dementia	F00–F03

Supplementary Table S2 - Missingness Summary and Exclusions:

Variable	Missing n	Missing %	Notes
TBSA (KOF)	0	0%	Complete
Burn mechanism	1	0.15%	Single excluded patient
ABSI	0	0%	Complete
Psychiatric coding	0	0%	Complete
Inhalation injury	0	0%	Complete

A total of 645 patients were admitted between 2014 and 2024. One patient was excluded due to missing burn mechanism data, resulting in a final cohort of 644 patients.

Supplementary Table S3. Annual Prevalence of Psychiatric Diagnoses (2014–2024)

Year	Total N	Psych N	Psych %	Non-Psych N	Non-Psych %
2014	49	11	22.4%	38	77.6%
2015	56	13	23.2%	43	76.8%
2016	57	16	28.1%	41	71.9%
2017	74	14	18.9%	60	81.1%
2018	83	17	20.5%	66	79.5%
2019	53	21	39.6%	32	60.4%
2020	59	18	30.5%	41	69.5%
2021	63	12	19.0%	51	81.0%
2022	54	21	38.9%	33	61.1%
2023	76	22	28.9%	54	71.1%
2024	21	12	57.1%	9	42.9%
